# Supplementary material for: First look at emergency medical technician wellness in India: Application of the Maslach Burnout Inventory in an unstudied population
Source: PLoS One. 2020 Mar 10;15(3):e0229954. doi: 10.1371/journal.pone.0229954 (PMC7064236; doi:10.1371/journal.pone.0229954)
Supplement: S1 File — (PDF) [file pone.0229954.s004.pdf]

STANFORD UNIVERSITY and GVK EMRI	Research Consent Form	IRB-41940
Protocol Director: Katie Koval		
Protocol Title: Prehospital Provider Wellness and Retention in India and the United States		

DESCRIPTION: The purpose of this survey is to better understand how you feel about your work at GVK EMRI.

TIME: The survey will take approximately 20-30 minutes to complete. You will not be paid to participate in this research study.

PARTICIPANT'S RIGHTS: Your participation in this study is completely voluntary. You do not have to complete this form if you do not want to. You have the right to refuse to answer particular questions. There are no right or wrong answers. Your willingness to fill out this survey will NOT impact your participation in the remainder of this course or your employment with GVK EMRI. **Please do not write your name or any identifying information on this paper. Please do not sign your name**. A copy of this form is for you to keep.

RISKS: The risks associated with this study are minimal but may include psychological discomfort or embarrassment should others somehow discover your identity. Supervisors will see your responses, but they will not be able to identify that it is your survey.

BENEFITS: The results of this survey may help create resources, programs, and policies to address EMT work satisfaction. The results of this research study may be presented at scientific or professional meetings or published in scientific journals. We cannot and do not guarantee that you will receive any benefits from this study.

CONSENT: Should you agree to participate, please mark an X in this box that you have read and understand the above information. Feel free to ask any questions before you sign an X. ☐

CONTACT INFORMATION:

Questions: If you have any questions, concerns or complaints about this research, its procedures, risks and benefits, contact the Protocol Director, **Katie Koval**, MD, katie.koval@gmail.com

Independent Contact: If you are not satisfied with how this study is being conducted, or if you have any concerns, complaints, or general questions about the research or your rights as a participant, please contact the Stanford Institutional Review Board (IRB) to speak to someone independent of the research team at 650-723- 5244. You can also write to the Stanford IRB, Stanford University, 3000 El Camino Real, Five Palo Alto Square, 4th Floor, Palo Alto, CA 94306.

Please answer the questions below to the best of your ability. Please read the questions carefully, and please answer honestly about how you feel.

How often:

0	1	2	3	4	5	6
Never	A few times a year or less	Once a month or less	A few times a month	Once a week	A few times a week	Every day

1._
2._
3._
4._
5._
6._
7.
8._
9.
10
11
12
13
14
15
16
17
18
19
20
21
22

Proprietary 22 Question Maslach Burnout Survey
https://www.mindgarden.com/117-maslach-burnout-inventory

23. _____ Patients appreciate the care I have provided to them.

In the past six (6) months how often have you:

24. _____ Experienced a patient death?
25. _____ Responded to a call involving a child victim?
26. _____ Responded to a call involving extreme violence or a rape victim?
27. _____ Responded to call that could be considered a disaster or terrorist attack?

Indicate how much you agree or disagree with the following statements:

0	1	2	3	4	5	6
Strongly disagree	Disagree	Somewhat Disagree	Neither Agree nor Disagree	Somewhat agree	Agree	Strongly Agree

28. ______ I feel satisfied with my job as an EMT.
29. ______ I am frustrated by the fact that I often am not allowed to care for patients to the fullest of my ability.
30. ______ I feel respected by my family for the work that I do
31. ______ I feel respected by the community for the work that I do
32. ______ I worry about being exposed to an infectious disease like HIV while at work
33. ______ I feel adequately trained to handle the patients I see
34. ______ I am not stressed with my monthly income and expenditure.
35. ______ I am treated poorly by emergency department personnel
36. ______ I am treated poorly treated by police
37. ______ My administrators have no idea of the typical work day for an EMT and what it takes to get the job done.
38. ______ I do not mind the irregular hours and the nightwork as an EMT
39. ______ The paperwork associated with my work is manageable
40. ______ The relationship with my pilot and fellow EMTs is supportive
41. ______ The equipment I work with is adequate to meet the healthcare needs of my patients
42. ______ I feel physically at risk during my job

43. What is your age in years? ________ years

44. Sex (circle one)

    Female Male

45. How many years have you worked as an EMT? ______ years

46. How many years do you plan to continue working as an EMT? _______years

47. In which state do you work? (circle one)
    1. Telangana
    2. Gujarat
    3. Uttarakhand
    4. Goa
    5. Tamil Nadu
    6. Karnataka
    7. Assam
    8. Meghalaya
    9. West Bengal
    10. Himachal Pradesh
    11. Chhattisgarh
    12. Uttar Pradesh
    13. Rajasthan
    14. Dadra & Nagar Haveli
    15. Daman & Diu

48. What is your work environment? (circle one)

    a. Urban b. Rural

49. What is the highest level of school education you have completed (circle one)
    a. Lower primary school
    b. Upper primary school
    c. SSC or Grade 10

d. HSCE or grad 12
e. University Degree (BSc or equivalent)
f. Post-Graduate Degree (MSc/MA, etc.)

50. What is your religion? (circle one)
    a. Hinduism
    b. Islam
    c. Christianity
    d. Buddhism
    e. Sikhism
    f. I am not religious
    g. Other _________________

51. What is your social status (circle one)
    a. Backwards caste
    b. Scheduled tribe
    c. Scheduled caste
    d. Other caste

52. What is your marital status? (circle one)
    a. Unmarried b. Married

52a. If married, does your spouse work outside of the home? (circle one)
Yes No

53. How many children do you have? (number) _______

54. Are you responsible for the daily care of someone? (circle one)
    a. Yes b. No

54a. If yes, (circle one)
    a. Sick family member
    b. Child
    c. Other___________________

55. Are you the financial breadwinner of your family? (circle one)
Yes No

55a. If yes, how many people are dependent on your income? ______ people

56. Do you work in your home state? (circle one)
Yes No

56a. If no, which state do you consider home? (circle one)

1. Andhra Pradesh (AP)
2. Arunachal Pradesh (AR)
3. Assam (AS)
4. Bihar (BR)
5. Chhattisgarh (CG)
6. Goa (GA)
7. Gujarat (GJ)
8. Haryana (HR)
9. Himachal Pradesh (HP)
10. Jammu and Kashmir (JK)
11. Jharkhand (JH)
12. Karnataka (KA)

13. Kerala (KL)
14. Madhya Pradesh (MP)
15. Maharashtra (MH)
16. Manipur (MN)
17. Meghalaya (ML)
18. Mizoram (MZ)
19. Nagaland (NL)
20. Odisha(OR)
21. Punjab (PB)
22. Rajasthan (RJ)
23. Sikkim (SK)
24. Tamil Nadu (TN)
25. Tripura (TR)
26. Uttar Pradesh (UP)
27. Uttarakhand (UK)
28. West Bengal (WB)

57. On average, how many hours of one-way travel does it take for you to see your family?
______ hours

58. On average, how many days per month do you get to spend time with your family?
_____ days / month

59. On average, how many patients do you see per 12 hour shift?
______ patients / 12 hour shift

60. On average, during the past month, how many hours have you worked as an EMT each week?
______ hours / week

61. During the past 1 month have you worked a shift longer than 13 hours? (circle one)
Yes No

61a. If yes, what was the longest shift that you worked? _____ hours

61b. If yes, how many shifts in the past 1 month have you worked that were longer than 13 hours?
____ shifts in the last month

62. When was the last time you were granted a holiday / leave away from work? (circle one)
a. In the last month
b. In the last 3 months
c. In the last 6 months
d. In the last year
e. I have not had a holiday for the last 1 year

63. Is the above leave in accordance with the policies with GVK EMRI? (circle one)
Yes No

64. If GVK EMRI could make one change to improve your work satisfaction, what would that be?
____
____

Thank you for your participation! Should any of these questions provoke negative emotions upon recalling traumatic cases, we encourage you to reach out to your supervisor / EME / colleagues and family and friends to discuss these events.
