# Supplementary material for: First look at emergency medical technician wellness in India: Application of the Maslach Burnout Inventory in an unstudied population
Source: PLoS One. 2020 Mar 10;15(3):e0229954. doi: 10.1371/journal.pone.0229954 (PMC7064236; doi:10.1371/journal.pone.0229954)
Supplement: Attachment — Submitted filename: Response to Reviewers.docx [file pone.0229954.s005.docx]

**Response to Comments**

Reviewer #1: Although an interesting, this study is not original nor unique and does not add significantly to the current literature on EMS burnout. As such I do not believe this manuscript should be published in Plos One, but instead should be submitted to a journal more specifically focused on EMS or Indian Health Care.

*Thank you for the feedback. We think this study is an important addition to the literature with attention to an often forgotten prehospital workforce. No published studies have measured burnout in Indian EMTs. With an estimated 17,000 ambulances in operation across India’s states and territories [1], this study has implications for a very large workforce in India alone. Additionally, few have examined this challenge in low- and middle-income countries. The field of prehospital care is incredibly diverse worldwide. Differing work environments, training qualifications, employer policies, and local culture all play a critical role in the wellness of EMTs. As such we feel this study examining India’s unique prehospital environment adds to our understanding of burnout worldwide. Additionally, many PLOS one articles have examined workforce wellness in a variety of populations worldwide [2-5]. As such, we think PLOS one would be a great home for our manuscript.*

1. *Subroto D, Desai R. Emergence of EMS in India. J Emerg Med Serv. 2017;42(4).*
2. *Eiche C, Birkholz T, Jobst E, Gall C, Prottengeier J (2019) Well-being and PTSD in German emergency medical services – A nationwide cross-sectional survey. PLoS ONE 14(7): e0220154.* [*https://doi.org/10.1371/journal.pone.0220154*](https://doi.org/10.1371/journal.pone.0220154)
3. *Alfuqaha OA, Alkawareek MY, Alsharah HS (2019) Self-evaluation and professional status as predictors of burnout among nurses in Jordan. PLoS ONE 14(3): e0213935.* [*https://doi.org/10.1371/journal.pone.0213935*](https://doi.org/10.1371/journal.pone.0213935)
4. *Useche SA, Montoro LV, Ruiz JI, Vanegas C, Sanmartin J, Alfaro E (2019) Workplace burnout and health issues among Colombian correctional officers. PLoS ONE 14(2): e0211447.* [*https://doi.org/10.1371/journal.pone.0211447*](https://doi.org/10.1371/journal.pone.0211447)
5. *Balayssac D, Pereira B, Virot J, Collin A, Alapini D, Cuny D, et al. (2017) Burnout, associated comorbidities and coping strategies in French community pharmacies—BOP study: A nationwide cross-sectional study. PLoS ONE 12(8): e0182956. https://doi.org/10.1371/journal.pone.0182956*

Page 2 Lines 1-20
Please put abstract into the standard format, with subheadings to include: Introduction, Methods, Results, Conclusion.

*Thank you. Completed.*

Page 2 Line 4
Need to add an introductory/transition sentence.
Need to state that previous studies have identified burnout as an issue for EMS professionals in other countries.

*Thank you. Completed.*

Page 3 Line 45
You also need to include data from Germany which showed that the dimensions emotional exhaustion and depersonalization were positively associated with the safety outcomes injury and safety compromising behavior. Baier N, Roth K, Felgner S2, Henschke C. BMC Emerg Med. 2018 Aug 20;18(1):24. doi: 10.1186/s12873-018-0177-2.
Burnout and safety outcomes - a cross-sectional nationwide survey of EMS-workers in Germany.
and
also include study from
Nirel N, Goldwag R, Feigenberg Z, Abadi D, Halpern P. Stress, work overload, burnout, and satisfaction among paramedics in Israel. Prehosp Disaster Med. 2008 Nov-Dec;23(6):537-46.

*Thank you for the additional references / supportive studies. Both of these have been added.*

Page 9 Line 133 -135
Please clarify
Other EMS studies on burnout that used the MBI reported data as a Mean with SD. S Suggest you do the same.

*Thank you. The reason we chose median with IQR instead of mean with SD was due to the non-normal distribution of our data. We feel this is a more statistically sound reflection of our population’s results. Below are the means for our data compared to medians for our data. If comparison data was normally distributed, comparison of our medians to their means would still be a fair comparison:*

*Median	EE 18	DP 5	PA 42*

*Mean. EE 19.69	DP. 6.13	PA 41.21*

Page 9 Table 3
Suggest you make this into 3 separate tables. Be sure to also include total number in each study for example India N= 327, Romania N=258 etc.
Table 3A Emotional exhaustion
Table 3B Depersonalization
Table 3C Personal accomplishment

*Thank you for this suggestion. We have tried both 3 separate tables and 1 combined table and found the division to be very repetitive with only 1 column differing between each table. We have remodeled the table and added the study sample sizes.*

Page 9 Table 3
Probable typo from Romanian study.
Not sure where you got your Romanian data since paramedic data from Romania study were not consistent with the Romanian manuscript "The best-recorded values are for paramedics (EE=0.63, DP=0.46, PA=5.01)"

*Thank you for pointing this out as this deserves some clarification. The Romanian study presents average MBI scores, not the cumulative scores reflected in our Table 3 which are summative scores. As opposed to average scores, summative scores were chosen to compare across populations based on the recommendation from the MBI survey manual. To enable comparison of apples to apples, we converted the average scores Popa et al presented to total scores with the appropriate MBI multiplier per category. For example, average depersonalization score for Romanian paramedics was 0.46. Depersonalization is a 5-question measure. Therefore 0.46 (average DP) X 5 = 2.3 (data presented in our table). We have added a footnote to the table to clarify this.*

Page 9 Table 3
Typo/Errors regarding data from Turkish study.
The Turkist study has mainly paramedic not EMTs.
Furthermore, the Paramedic sub scores were 8.48 ± 3.91 13.57 ± 5.48 9.56 ± 4.87, not the ones you show.

*Again, thank you. You have highlighted a need for clarification. However, we did not find the subscores you refer to 8.48, 13.57, etc. In the Deniz et al study that we cite, the authors used a different scoring system than the typical MBI— page 297, 2nd paragraph of Material & Methods – they calculate their MBI scores based on a Likert scale of 0- 4 versus the MBI version we used that has a scale 0-6. As a result, we proportionally present their data using the same denominator. For example, their ambulance personnel score 11.594 on emotional exhaustion out of a maximum of 36 points as denominator (9 questions). The denominator for emotional exhaustion for most MBI surveys including ours is 54-- which adjusting proportionally results in a score of 17.39 (our Table 3). We have added a footnote to clarify our adjustment.*

*Deniz et al define their population as ambulance service workers. We did not find further specificity in the paper that categorizes them as paramedics. However, we have changed the population descriptions to better reflect the exact terminology used in each study, and hopefully better reflect the diversity of prehospital personnel.*

Page 9 Table 3
Most of the other studies gave results as a mean with standard deviation.
Thus, to better compare your study to the others suggest you include the mean and SD given in the other studies for Emotional exhaustion, Depersonalization, and Personal accomplishment.

*Thank you. See response to Page 9 Line 133 -135 above*

Page 10 Lines 150-153
If possible do not document any results as a p value, but instead use an Odd Ratio with 95% CI. Currently reads “Younger EMTs experienced more burnout (p=0.034).”
Should read “Younger EMTs experienced more burnout (OR 0.90, 95%CI=0.81 – 0.99)”

*Thank you. Completed.*

Page 12 Line 191
Expand discussion on why Romanian data was different.
“Burnout levels are lower than those found in other studies [30] and the results might be correlated with the fact that paramedical staff is army-enrolled and must pass periodical psychological examinations ... not mandatory for any other of the surveyed”

*Thank you. Completed.*

Page 14 Line 231
Please briefly expand the discussion with two sentences on sleep deprivation.
Please add that another potential cause of burnout includes sleep deprivation and 24 hr shifts and whether this is an issue in your study population and include:
Patterson PD, Weaver MD, Frank RC, et al Association between poor sleep, fatigue, and safety outcomes in emergency medical services providers. Prehosp Emerg Care. 2012 Jan-Mar;16(1):86-97. doi: 10.3109/10903127.2011.616261. Epub 2011 Oct 24.
and
Bennett P, Williams Y, Page N, et al. Associations between organizational and incident factors and emotional distress in emergency ambulance personnel. Br J Clin Psychol. 2005;44(Pt 2):215-26.
And your current reference #25
Popa F, Raed A, Purcărea VL, Lală A, Bobirnac G. Occupational Burnout levels in
326 Emergency Medicine – a nationwide study and analysis. J Med Life. 2010;3(3):207–15.

*Thank you. Our survey touched on this topic, but this was not a focus for this paper. Our survey asked 1 question about perception of schedule “I do not mind the irregular hours and the nightwork as an EMT” with Likert responses. 44% of EMTs agreed with this statement. Additionally in single variate regression, responses to this question were not differently distributed between those EMTs who were and were not experiencing burnout (OR 0.69, 95% CI=0.41-1.16, p=0.166).*

*"Some have found that sleep deprivation contributes to burnout, suggesting that changes to EMT schedules and hours may be a modifiable risk factor to mitigate burnout [50,51]. However in our study, EMT’s experiencing burnout did not mind irregular work hours or night shifts any more than EMTs who had low levels of burnout (OR 0.69, 95% CI=0.41-1.16, p=0.166).  This may be a reflection of the 12-hour shift length that is standard for the organization nationwide.”*

Reviewer #2: Overall this paper does add knowledge to the EMS literature base. Little is known about the rates of burnout in India. This is a very relevant topic - provider wellness is becoming incredibly important to high functioning EMS systems especially in the US where suicide rates are climbing.

I do feel that the paper is placing substantial emphasis on secondary outcomes and making causative conclusions about data found on analyzing the non-primary data points. This can be somewhat misleading to readers as the strength of this evidence is diminished.

*Thank you. As you are aware, the area of EMT wellness in India is unstudied. The primary outcome of this study was focused and powered to provide the prevalence rate of burnout. We feel it is equally important to explore any predictors of burnout that could shed light on the problem identified and see this study as hypothesis generating. We have reviewed the Discussion and Conclusions of this paper to ensure that we are not overstating insights that can be drawn from our study.*

16: You don't define LMIC until later in the paper.

*Thank you. This has been corrected.*

64: The US reader would be more familiar with the term EMT-Basic, although national standards have been revised to just "EMT" for this level of care.

*Thank you. We agree with this suggestion and have made that change.*

*“Healthcare Sector Skill Council.” (2013). Emergency Medical Technician-Basic. Retrieved from Qualification Pack - National Occupational Standards (QP-NOS) website: http://www.healthcare-ssc.in/national-occupational-standards-page-193*

65: You mention at the end of this paper that your sample was a convenience sample - can you explain here how these EMTs were selected for study? The way the classes and subjects were chosen may introduce substantial bias into the data.

*Thank you. More information has been added to Methods and an additional phrase to the Limitations section.*

*Methods: “Surveys were distributed to a convenience sample of Indian EMT-Basics working for the largest ambulance service in India, GVK EMRI. EMT-Basics with this employer receive 52 days or 450 hours of theory and skills training from their employer prior to working on the ambulance, more than the national requirements [36]. Participants were approached during regularly scheduled continuing medical education courses conducted by their employer. EMTs presenting for continuing medical education between July-November 2017 from Gujarat, Karnataka, and Telangana were eligible to participate. EMTs typically travel twice annually to attend courses in Hyderabad and were scheduled to attend classes prior to determination of study dates.  During study windows, all classes were approached for participation.”*

*Limitations: “The strength of this study is also limited by a convenience sample which may introduce selection bias.”*

83-85: It's not clear why these studies were not analyzed. Is that part of the instructions in the MBI or a decision of the authors? If the latter, that's about 5% of your data set being excluded; there may be enough data to alter the statistical significance of some of your results.

*Thank you. This has been clarified. 296 surveys were able to be averaged and 280 had complete MBI scores. Both the averages and summative scores are discussed in the paper.*

*“As advised by MBI survey creators, if respondents omitted more than one answer per MBI component, their survey was not averaged, and summative MBI scores required all 22 questions to be completed.”*

85-86 your later discussion mentions the difficulties with translation of the surveys. I think you need to provide much more detail as to how this occurred. Was the translation done by someone blinded to your study? Were they an author on this paper? Was it a translation or an interpretation? Was the product reviewed by others?

*Thank you. We have added more details to the methods.*

“*Surveys were translated and then reverse-translated by a second native speaker. Translators were independent of study. The two translations were then reconciled and interpreted with study staff and a third native speaker to ensure the essence of questions was reflected.”*

102: ambulance pilot implies aeromedical services

*Thank you. This is the terminology used within GVK EMRI but for clarity has been changed to “driver.”*

115-117: I find this incredibly interesting as degree requirements for EMS providers in the US is a very controversial topic. This is an excellent jumping point for further study based on your data.

*Thank you, we will discuss and consider!*

118-119: The western reader may not understand these examples given

*That is definitely true. However for the Indian context, we think this is still an important marker of socioeconomics and culture that could introduce variability / confounding and was an important variable to analyze and include.*

Table 2:
No and Little evidence of burnout seem to be vastly different concepts in the heading

*Thank you. We have changed the column headings to be more directly reflective of the MBI wording. Table 2 now reads “High Degree of Burnout” and “Low to Moderate Degree of Burnout.”*

It's not clear how the P values are tied to the adjacent data. Is it only presented for significant findings?

*All p values are presented, not just significant values. The gray shading in the table was an effort to group analyses together. For example, p=0.001 reflects the difference between all 3 compared states. The p values in the table have been moved to be clearer as to what is being compared.*

Table 3:
Why was USA physicians picked as a line for this table? You mention Indian physicians elsewhere in the paper as a reference for burnout but don't include them in the table.

*Unfortunately, there is little to no data for the MBI in a comparable population in the US, so US physicians were originally included for context. But in light of your point, this data has been removed.*
*Indian physicians were not included in Table 3 because that study did not include a full MBI survey which prevented us from directly comparing those populations with an unequal score / denominator. However because of the importance of this comparison, we elaborated on this analysis separately in the Discussion section and included it in the supplementary tables to demonstrate that analysis using same scoring system of the abbreviated MBI.*

150: Age is a continuous variable. How did you define "younger"?

*Thank you. We have changed the phrasing in Results and Discussion to reflect your point.*

157: Are these questions answered part of the standardized survey instrument or was this something additional added as part of the study?

*Demographics are not part of the MBI but were added by our study team. MBI only includes questions 1-22 on the survey instrument. The full survey is included as a supplemental submission.*

16, 17 170, 177: 223-etc I dont think your data can support this causality you imply. You show association. Theoretically the decision to change professions could cause the results on their survey, no way to tell the difference in a cross-sectional study such as this. Also this doesn't belong in the analysis/methods section.

*Thank you. The very last statement in this paragraph, which was an interpretation of the data, has been removed leaving only results. The language here and in the Conclusion been changed to reflect association and not causality.*

224: need a reference for this.

*Thank you. Reference has been added.*

*Hann M, Reeves D, Sibbald B. Relationships between job satisfaction, intentions to leave family practice and actually leaving among family physicians in England. Eur J Public Health. 2011;21(4):499–503*

Grammar:
Overall needs thorough read over for grammatical errors. Some sentences / sections start with words such as "yet" and "but" which is awkward and needs revision.

*Thank you. Global review and revisions completed.*

Reviewer #3: Thank you for diving into a topic that is rarely discussed, provider well being. It is very important to improve their job satisfaction in order to better serve the public. Please consider this suggestions for your manuscript.

Methods section:
How ere the study staff trained to administer the survey? Did they clarify questions for the participants? If so, this could also be a limitations because different staff might define a question very differently.

Who translated the survey and how did you ensure it was a meaningful translation? There was mention of an "initial" translation in the limitations sections.

*Thank you. More details have been added in Methods to help clarify these concerns. But no, EMT instructors for courses read the consent aloud, answered logistical questions about how to complete the survey and then were asked to leave the room prior to the actual completion of the survey. The study staff proctoring the surveys did not speak the native language and could not clarify translation questions.*

*“EMT instructors read the informed consent aloud, answered questions about how to complete survey, and were then asked to leave the room during survey administration to ensure EMTs anonymity and privacy. Independent study staff proctored, collected, and analyzed the surveys.”*

*"Surveys were translated and then reverse-translated by a second native speaker.  Translators were independent of study. The two translations were then reconciled and interpreted with study staff and a third native speaker to ensure the essence of questions was reflected.”*

Line 16 - You should spell out "LMIC" in the abstract

*Thank you. Completed.*

Line 26 - Is it "development community" or "developing communities"?

*Thank you. We had intended it to be as written “development”— i.e. those who work in public health and global development.*

Line 29 - Would add (WHO) after "World Health Organization" in case you need to repeat the term.

*Thank you. Completed.*

Line 33 - Consider changing sentence to: "...continuous to be a crisis and India is no exception."

*Thank you. Completed.*

Line 35 - Consider changing to "...improve quality of care reducing the need for and cost..."

*This changes the meaning of the sentence to something different. We want to demonstrate the multiple benefits by a focus on wellness.*

Line 40 - change "and" to a comma: "...providers .worldwide, particularly among emergency ..."

*Thank you. Completed.*

Line 42-43 - Add "the" in front of "health care system"

*Thank you. Completed.*

Line 54 - Consider change to "Having a better understanding of burnout rates..."

*Thank you. Completed.*

Line 59 - Consider change to "...demanding conditions and the particular stresses of emergency care.”

*Thank you. Completed.*

Line 64 - Consider change to "Indian basic level EMTs..."

*Thank you. Completed.*

Line 67 - Change "As" to "Since"

*Thank you. Completed.*

Line 96-100 - This is kind of a long sentence with multiple uses of "and" - Consider re-writing this sentence or breaking it up.

*Thank you. Completed.*

Line 115-116 - Change sentences to "...almost evenly between rural and urban environments..." "The majority of sampled EMTs identified as ..."

*Thank you. Completed.*

Line 194 Change to "...has a strong EMT recognition program..."

*Thank you. Completed.*

Line 196-197 - Change to "...very young in India so recognition and respect for the role of the EMT outside the organization (i.e. in the family, community and the health care as a whole) may be lagging."

*Thank you. Completed.*

Line - 200-201 - Consider change to "Significant associations between administrator support, emotional exhaustion scores and burnout have also been demonstrated by others [34-36].

*Thank you. Completed.*

Line 204 - Consider change to "... personnel had the strongest correlation with burnout.”

*Thank you. Completed.*

Line 204 - Consider change to "A study of EMTs in the Netherlands also demonstrated a significant..." Line 208 delete “while"

*Thank you. Completed.*

Line 213 - Change "more than two times as likely" to "more than twice as likely"

*Thank you. Completed.*

-------

I hope this recommendations are useful to you. Thank you for allowing me to review this manuscript.

Reviewer #4: GENERAL
Please insert a space between the last word and [reference].

*Thank you. Completed.*

ABSTRACT
Please follow PLOS ONE guidelines for abstract structure and include appropriate sub-headings.

*Thank you. Completed.*

INTRODUCTION
The readers would benefit from an early clarification of definitions and differences between stress and other associated terms. Stress is experienced on a continuum from eustress to burnout syndrome (BOS). In extreme cases, providers may develop signs and symptoms of posttraumatic stress disorder (PTSD).

May consider including some discussion on stress coping mechanisms and their transition for adaptive to maladaptive.

Line 37-39: Please provide reference(s). I would favor clarifying how the definition has evolved over time. The concept of BOS, as first described by Freudenberger in 1974, refers to a protracted course of distress in which one is unable to cope with stressors over an extended period of time, leading to depletion of the body’s defenses and ultimately physical and emotional exhaustion. In 1996, this concept was further refined and BOS was defined as a syndrome of emotional exhaustion, depersonalization, and reduced personal achievement.

*Thank you. We have added more information about burnout, its history, and a brief allusion to the complicated interplay of depression, PTSD, stress and burnout.*

*“The concept of burnout popularized by psychologist Freudenberger in 1974 was initially described as “becoming exhausted by making excessive demands on energy, strength, or resources” resulting in a physical and behavioral syndrome from the workplace. His initial study involved workers at a demanding free clinic in New York City [7].  Maslach et al standardized the measurement of burnout in the late 1970’s refining its definition to a state of exhaustion, cynicism, and diminished professional efficacy that results from long-term involvement in work situations that are emotionally demanding and is a critical factor in employee retention and performance [8]. More recent research has focused on clarifying the relationships between stress, burnout, depression, and post-traumatic stress disorder to better understand their distinctions as well as the personality factors and adaptive mechanisms that are protective [9–11].”*

Line 40: Please provide some statistics to clarify the scope of the problem.”

*Thank you. This has been added.*

*“There is increasing evidence that burnout is common among a range of healthcare providers, however evidence is limited in the prehospital workforce. A US-based MBI study of physicians demonstrated emergency physicians with the highest level of burnout at almost 70% compared to physicians of other specialties [7].  Previous research in Indian physicians of all specialties demonstrates a burnout prevalence of 35%-71% [8–10].  Less than fifty studies have examined burnout in the prehospital realm of emergency medical technicians (EMTs).  Only a handful of these include EMTs in LMIC.  Studies of EMTs internationally, including varied burnout assessments such as the Copenhagen Burnout Inventory, report work-related burnout rates ranging from 19%-84% [11–16].  No published studies have measured burnout in Indian EMTs.”*

Lines 43-53: Each of these references refers to physicians and nurses; the statistics, outcomes, and variables that affect these groups may be different than EMTs. Please provide appropriate references for your target study group (EMTs).

*Thank you. References have been reorganized and added with data specific to EMT populations.*

*Crowe RP, Bower JK, Cash RE, Panchal AR, Rodriguez SA, Olivo-Marston SE. Association of Burnout with Workforce-Reducing Factors among EMS Professionals. Prehospital Emerg Care. 2018;22:229–36]*

*Baier N, Roth K, Felgner S, Henschke C. Burnout and safety outcomes-a cross-sectional nationwide survey of EMS-workers in Germany. BMC Emerg Med. 2018;18(1):24.*

METHODS:
Has the MBI been validated in an Indian cohort? If so, please state and provide reference.

*Thank you. The MBI has not been formally validated in India. We have added this to the Limitations section.*

*“While the MBI has been used before in Indian healthcare populations [8,10], it has not been officially validated in India.”*

Line 64: Clarify what constitutes a “basic” EMT? What is this opposed to?

*Thank you. Training levels / qualifications of EMTs internationally are diverse. But generally follows international delineation between “EMT Basics” and “EMT Advanced / Paramedics.” We have added more details on the training requirements of our population of EMT basics and a reference to the minimum requirements for India as a whole.*

*“Healthcare Sector Skill Council.” (2013). Emergency Medical Technician-Basic. Retrieved from Qualification Pack - National Occupational Standards (QP-NOS) website: http://www.healthcare-ssc.in/national-occupational-standards-page-193*

Please specify that the sample is a convenience sample. Was any personal information collected or stored?

*Thank you. The English version of the survey is included as a supplement. Basic demographic information including age, sex, marital status, religion, and state of work were collected without other identifying information like date of birth, EMT numbe,r or name.*

*“Surveys were distributed to a convenience sample of Indian EMT-Basics (as opposed to EMT-Advanced) working for the largest ambulance service in India, GVK EMRI. EMT-Basics with this employer receive 52 days or 450 hours of theory and skills training from their employer prior to working on the ambulance, more than the national requirements [36].”*

How/where was the survey announced or advertised? Ideally the survey announcement should be published as an appendix.

*Thank you. There was not an announcement of the survey to recruit participants. EMTs were approached as part of their semi-annual CME classes. During CME class breaks, a study coordinator entered the room and the consent was read aloud by EMT instructors (native speakers).*

*“Participants were approached during regularly scheduled continuing medical education courses conducted by their employer. EMTs presenting for continuing medical education between July-November 2017 from Gujarat, Karnataka, and Telangana were eligible to participate. EMTs typically travel twice annually to attend courses in Hyderabad and were scheduled to attend classes prior to determination of study dates.  During study windows, all classes were approached for participation.”*

Were any incentives offered (eg, monetary, prizes, or non-monetary incentives such as an offer to provide the survey results)?

*Thank you. No incentives were provided.*

*“Incentives were not offered for participation. Respondent names were not collected to maintain anonymity. EMT instructors read the informed consent aloud, answered questions about how to complete survey, and were then asked to leave the room during survey administration to ensure EMTs anonymity and privacy. Independent study staff proctored, collected, and analyzed the surveys.”*

Indicate whether any methods such as weighting of items or propensity scores have been used to adjust for the non-representative sample; if so, please describe the methods.

*Thank you. Weighting / propensity scores were not used. One of the reasons we feel this study is so valuable is because it is the first in India among EMTs and among the first in a LMIC. As a result, there is no normative sample to adjust to / for. We do not know the defining or important predictors of burnout and as a result did not feel that propensity scores would be appropriate.*

RESULTS / DISCUSSION
Line 138-141: This is an interesting observation. Why do you think that is? Please include in discussion.

*Thank you. This discussion is included in new lines 288- 293*

Line 150: Why do you think younger EMTs were more likely to experience burnout? This is similar to what has been observed in some nursing populations. Please include in discussion.

*Thank you. This discussion is included in new lines 314-319*

Line 151: Why do you think women were more likely to experience burnout? This is the opposite of what has been observed in some nursing populations. This has been observed in nurses as well. Please include in discussion.

*Thank you. See Below*

Line 174-176: Didn’t you state that these findings were not significant after adjusting for controlling factors (line 152-153).

*State and gender when analyzed individually in univariate analysis appeared to be predictive, but when controlling in multivariate regression for age, state, gender, etc (Table 4) state and gender were NOT predictive of burnout. The other factors like physical risk, perceived lack of respect, and age better explained burnout than state or gender. As a result, we did not delve into the relationship between women and burnout (comment above) as it was not found to be significant.*
